# Maternal sociodemographic characteristics and the use of the Iowa Infant Attitude Feeding Scale to describe breastfeeding initiation and duration in a population of urban, Latina mothers: a prospective cohort study

**DOI:** 10.1186/1746-4358-8-7

**Published:** 2013-07-08

**Authors:** Katherine E Holbrook, Mary C White, Melvin B Heyman, Janet M Wojcicki

**Affiliations:** 1 Pediatric Gastroenterology, Hepatology, and Nutrition, Department of Pediatrics, University of California San Francisco, 500 Parnassus Avenue, MU 4-East, San Francisco, CA, 94143-0136, USA; 2 School of Nursing, Department of Community Health Systems, University of California San Francisco, 2 Koret Way Suite #N-505, San Francisco, CA, 94143-0608, USA

**Keywords:** Breastfeeding, Latina, Lower socioeconomic status, Obesity

## Abstract

**Background:**

The World Health Organization recommends exclusive breastfeeding until 6 months of age. Maternal attitudes toward infant feeding are correlated with chosen feeding method and breastfeeding duration. The Iowa Infant Feeding Attitude Scale (IIFAS) has been used to assess attitudes towards breastfeeding prenatally and is predictive of breastfeeding decisions in certain population groups.

**Methods:**

In a cohort of pregnant Latina women recruited from two hospitals in the San Francisco Bay Area (n=185), we administered the IIFAS prior to delivery. Information regarding feeding choice, maternal sociodemographic information, and anthropometrics were collected at 6 months and 1 year postpartum. Analysis of predictors for breastfeeding initiation, breastfeeding at 6 and 12 months and exclusive breastfeeding at 6 months were evaluated using multivariate logistic regression adjusting for potential confounders.

**Results:**

In our cohort of Latina mothers, breastfeeding a previous infant was associated with breastfeeding initiation (OR 8.29 [95% CI 1.00, 68.40] p = 0.05) and breastfeeding at 6 months (OR 18.34 [95% CI 2.01, 167.24] p = 0.01). College education was associated with increased exclusive breastfeeding at 6 months (OR 58.67 [95% CI 4.97, 692.08] p = 0.001) and having other children was associated with reduced breastfeeding at six months (OR 0.08 [95% CI 0.01, 0.70] p = 0.02). A higher IIFAS score was not associated with breastfeeding initiation, breastfeeding at 6 or 12 months or exclusive breastfeeding at 6 months of age.

**Conclusions:**

Initial choices about breastfeeding will likely influence future breastfeeding decisions, so breastfeeding interventions should specifically target new mothers. Mothers with other children also need additional encouragement to maintain breastfeeding until 6 months of age. The IIFAS, while predictive of breastfeeding decisions in other population groups, was not associated with feeding decisions in our population of Latina mothers.

## Background

Breastfeeding is associated with significant maternal and infant health benefits and is promoted as the optimal form of infant feeding by the American Academy of Pediatrics (AAP) and the World Health Organization (WHO) [1]. The AAP and the WHO recommend exclusive breastfeeding for the first 6 months of life [2,3]. The WHO further recommends continuing breastfeeding with appropriate complementary foods for at least 2 years [3]. Exclusive breastfeeding is defined as the infant receiving only breast milk with no supplementation of water, juice, formulas or other liquids and foods, with the exception of vitamins or minerals, oral rehydration salts and medications [4]. Compared with supplemented breastfeeding, exclusive breastfeeding has been shown to provide greater protection for infants against lower respiratory tract infections, diarrhea, acute otitis media, atopic dermatitis, and childhood obesity and is associated with improved cognitive development [1,5-8].

Despite the AAP and WHO recommendations, in the U.S. only 13.8% of infants are exclusively breastfed at 6 months; in the Hispanic or Latino population, the percentage is lower at 13.4 [9]. A variety of factors influence a woman’s decision or ability to initiate and continue breastfeeding, including personal characteristics such as age, race, education and employment status, attitudinal and intrapersonal characteristics, hospital policies and intrapartum experience, sources of support, childcare status of the infant, and breastfeeding interventions [10-12].

The Latino population—individuals of Hispanic, Caribbean or Latin American descent from countries where Spanish is the primary language—comprises 16% of the U.S. population, and accounted for more than half of the growth in the total U.S. population between 2000 and 2010 [13]. As this group continues to grow in the U.S., understanding the sociodemographic factors that may influence breastfeeding initiation and duration in this population is important. Based on Californian hospital-based surveys administered 24–48 hours postpartum, Hispanic breastfeeding initiation rates are high at 90.5%, and exclusive breastfeeding rates are 48.3% [14]. In contrast, the national average for breastfeeding initiation in Hispanic or Latino mothers is lower at 80.3% [12]. In California, at 1 month postpartum, however only 72.5% of Hispanic mothers breastfeeding and 37.5% are exclusively breastfeeding. At 3 months postpartum the percentage is even lower with 51.2% of Hispanic mothers are breastfeeding and 21.7% are exclusively breastfeeding [15].

Risk factors for sub-optimal feeding in this population include increased acculturation to the US culture and pre-pregnancy obesity. Recent data show that foreign-born women are more likely to initiate breastfeeding than mothers acculturated into mainstream US culture [14,16-18] and that less acculturated women are more likely to exclusively breastfeed [19]. Additionally, for pregnant Hispanic women, pre-pregnant obesity was negatively associated with breastfeeding initiation and duration [20].

The Iowa Infant Feeding Attitude Scale (IIFAS) [21] was developed to assess maternal attitude toward infant feeding which is indicated as a predictor for feeding method choice and, among breastfeeding women, the duration of breastfeeding [22,23]. The scale has been used to assess both attitudes toward breastfeeding and to predict breastfeeding outcomes. The IIFAS has acceptable reliability and validity and has been tested in various populations including prenatal and postpartum women, low income women, fathers, students, health providers and health visitors although not in Latina women [24-29]. The scale has been adapted for use in Romania [30] and Croatia [31], and with Taiwanese [32], Jordanian, and Syrian women [33]. The IIFAS has also been used as a foundation for developing new scales for Chinese women [34] and Saudi women [35].

The purposes of this study are to explore sociodemographic factors that affect breastfeeding initiation and duration as well as to describe the association between attitudes towards breastfeeding (as outlined in the IIFAS) and actual breastfeeding initiation and duration.

## Methods

This study represents an analysis of maternal sociodemographic factors and attitudes toward breastfeeding initiation and duration, and exclusive breastfeeding from a longitudinal Latino mother-child cohort previously described [36,37]. Data for this study were included only of women who had complete data on initiation of breastfeeding at baseline and were seen for follow up at 6 and/or 12 months. Data not gathered in the primary study were gathered from chart review when available. A variety of maternal characteristics were examined, based on previous studies and biological plausibility.

### Participants

Exclusion criteria for participants included mothers who were using illegal drugs or alcohol, had pre-pregnancy diabetes or gestational diabetes mellitus treated with insulin, had polycystic ovaries, or eating disorders, or who anticipated having any health problems that would prevent breastfeeding as described previously [36,37]. Women were also excluded if breastfeeding data were unavailable at both the 6 and 12 months follow up interviews. Infants at delivery were excluded if they had special care needs, chronic disease, or Apgar scores less than 7 at 5 minutes. We excluded 16 of the 201 mother-infant pairs based on the exclusion criteria listed above, loss to follow up, or desire to discontinue participation.

### Procedures

Following informed consent and collection of sociodemographic information, the IIFAS was administered. The IIFAS is a 17 question survey to assess maternal attitudes toward breastfeeding. It has previously been tested for reliability and validity in a series of studies of primarily White, middle-class, English-speaking women [21]. Studies using the IIFAS report adequate predictive validity and internal consistency with the Cronbach’s alpha ranging from 0.79 in Scotland [26] to 0.86 in the United States and Ireland [21,28] and 0.89 in Scotland [25]. Total attitude scores range from 17, reflecting positive formula feeding attitudes, to 85, indicative of attitudes that favor breastfeeding. Maternal height and weight were measured or recorded from medical charts. Medical history, including mental health history, was assessed by chart review, questionnaire and mental health screening tools. Additionally, at 6 and 12 months postpartum, sociodemographic information and participant weight were collected.

All questions and instruments were administered in either English or Spanish by trained research assistants. All procedures were approved by the Committee on Human Research at University of California, San Francisco, and the Institutional Review Board at San Francisco General Hospital (IRB 11–06163).

### Statistical analysis

The main outcomes of interest were breastfeeding initiation, any breastfeeding at 6 months, exclusive breastfeeding at 6 months, and any breastfeeding at 12 months. Chi-square tests of association and analysis of variance tests were applied to evaluate the relationships among sociodemographic factors and breastfeeding initiation and duration, exclusive breastfeeding at 6 months, and breastfeeding at 12 months postpartum. Exclusive breastfeeding was defined as feeding the infant only breast milk without providing any other liquids or foods, although vitamin/mineral drops or medications were permitted according to the WHO’s definition of exclusive breastfeeding [4]. We defined statistical significance as *p* < 0.05. Variables with a *p* < 0.10 in bivariate analyses were analyzed using logistic regression. The main predictor of interest was the response to the IIFAS. The IIFAS was analyzed both as a continuous variable (total score) and as a dichotomous variable (low score versus high score) for the purpose of bivariate analyses, as analyzed in previous studies [38,39,43]. Mothers with a total score less than the median were assigned to the low score group, while those with a total score greater than or equal to the median were assigned to the high score group (median = 68).

Whether a participant had any number of children prior to this pregnancy was added into the multivariate models to control for the relationship between having children and previous breastfeeding. Having other children was defined as having given birth to another child, regardless of if the child still lived at home with the study participant. Children living in the participant’s home but who were not biological children of the study participant were not included in this variable. Years in the country and birth country were not included together with primary language in the multivariate models owing to the correlation among these variables; only the variable *primary language* was included as a predictor. Only participants with complete data on the selected variables were included in the multivariate models. Data were entered in Excel and subsequent analyses were conducted using SPSS version 19 statistical software (IBM Corporation, Armonk, NY).

## Results

Of the 201 women enrolled prenatally, 185 were included in the analyses after delivery, 185 were included at 6 months after birth and 170 were included at one year after birth. Five participants were not included at delivery owing to the development of insulin-treated gestational diabetes mellitus. At 4–6 weeks postpartum an additional 4 participants were excluded due to loss to follow up (n = 2), maternal health contraindications for breastfeeding and inability to participate (n = 1), and participant desire to drop out (n = 1). At 6 and 12 months postpartum, a further 7 participants were excluded due to loss to follow up.

### Demographic factors

In this restricted sample of our original cohort [36,37], mean maternal age was 26.3 ± 5.2 years (Table 1). While 93.0% were foreign born, the majority were Mexican-born (55.4%). Most spoke Spanish as a primary language (93.5%), and almost half (53.2%) had spent 5 or fewer years in the United States. The majority were partnered (83.7%); partnered was defined as being married, cohabitation with a partner, or being in a relationship. Of those who answered the question, most (n = 124, 67%) were not employed. Most had an education of high school or less (n = 143, 78.6%). The majority (n = 171, 92.4%) were enrolled in a WIC program and of those who had other children, 92.0% previously breastfed. At baseline, 34.7% (n = 61) women scored positive for depression on at least one of the three depression screening instruments. The average maternal BMI was 26.1 kg/m^2^ prenatally, 29.0 kg/m^2^ at 6 months and 28.8 kg/m^2^ at 12 months postpartum. Prior to pregnancy, 33.5% were overweight and 19.2% were obese based on chart review.

**Table 1:** Maternal sociodemographic characteristics among 185 participants

**Characteristic**	**n (%)**^**a **^**or mean±SD**
Maternal age in years	26.3±5.2
Country of birth	
USA	12 (6.5)
Mexico	102 (55.4)
Central America	62 (33.7)
Other	8 (4.3)
Primary language	
English	12 (6.5)
Spanish	173 (93.5)
Years living in the US	
≤1	25 (14.6)
> 1 and ≤ 5	66 (38.6)
> 5 and ≤10	49 (28.7)
> 10	31 (18.1)
Years in US for foreign born	6.4±5.9
Marital status^b^	
Partnered	154 (83.7)
Not partnered	30 (16.3)
Any employment	
Yes	61 (33.0)
No	124 (67.0)
Education	
High school or less	143 (78.6)
Some college	30 (16.5)
College	5 (2.7)
Post college	4 (2.2)
WIC participation^c^	
Yes	171 (92.4)
No	14 (7.6)
Other children	
Yes	99 (53.5)
No	86 (46.5)
Previously breastfed^d^	
Yes	92 (92.0)
No	8 (8.0)
Depressive symptoms at intake^e^	
Yes	61 (34.7)
No	115 (65.3)
Depressive symptoms 4–6 weeks postpartum	
Yes	30 (16.6)
No	151 (83.4)
BMI^f^	
Pre-pregnancy	26.1±5.5
6 months postpartum	29.0±6.5
12 months postpartum	28.8±6.3
Pre-pregnancy BMI category^g^	
Normal (< 25)	79 (47.3)
Overweight (≥25 or <30)	56 (33.5)
Obese (≥30)	32 (19.2)

^a^ n and percentages represent those who answered the question.

^b^ Partner status identified by women and included single but living with a partner.

^c^ Women, Infants and Children.

^d^ Calculated among those who had previous children (n=100).

^e^ Participants scoring positive on at least one of the three depression instruments administered.

^f^ Body Mass Index, calculated in kg/m^2^.

^g^ Centers for Disease Control and Prevention. *Body Mass Index*. Division of Nutrition, Physical Activity and Obesity, Atlanta, GA, 2011. http://www.cdc.gov/healthyweight/assessing/bmi/index.html.

### Breastfeeding initiation

Of 185 women, 177 (95.7%) initiated breastfeeding (Table 2). No significant differences were found in maternal age, country of birth, primary language, years in the U.S., marital status, employment status, education level, WIC participation, having other children, presence of depressive symptoms, BMI and BMI category between participants who initiated breastfeeding and participants who did not. Women who previously breastfed were much more likely to initiate breastfeeding than women who had not previously breastfed (OR 9.9, p = 0.05). Additionally women who initiated breastfeeding had a significantly higher average score on the IIFAS compared with women who did not (67.7 versus 61.6, p = 0.02) (Table 2).

**Table 2:** Maternal sociodemographic characteristics of participants who initiated breastfeeding versus those who did not initiate breastfeeding

**Characteristic**	**Initiated breastfeeding n (%) or mean**±**SD (n = 177)**	**Did not initiate breastfeeding n (%) or mean**±**SD (n = 8)**	**Total n (%)**^**a **^**or mean**±**SD n = 185**	**p value**
Maternal age in years	26.3±5.3	27.5±4.2	26.3±5.2	0.52
Country of birth				0.47
USA	12 (6.8)	0 (0.0)	12 (6.5)	
Mexico	96 (54.5)	6 (75.0)	102 (55.4)	
Central America	60 (34.1)	2 (35.0)	62 (33.7)	
Other	8 (4.5)	0 (0.0)	8 (4.3)	
Primary language				0.58
English	12 (6.8)	0 (0.0)	12 (6.5)	
Spanish	165 (93.2)	8 (100.0)	173 (93.5)	
Years living in the US	6.3±5.8	8.6±7.4	6.4±5.9	0.28
≤1	24 (14.7)	1 (12.5)	25 (14.6)	
> 1 and ≤ 5	64 (39.3)	2 (25.0)	66 (38.6)	
> 5 and ≤10	47 (28.8)	2 (25.0)	49 (28.7)	
> 10	28 (17.2)	3 (37.5)	31 (18.1)	
Marital status^b^				0.62
Partnered	147 (83.5)	7 (87.5)	154 (83.7)	
Not partnered	29 (16.5)	1 (12.5)	30 (16.3)	
Any employment				0.53
Yes	58 (32.8)	3 (37.5)	61 (33.0)	
No	119 (67.2)	5 (62.5)	124 (67.0)	
Education				0.48
High school or less	137 (78.3)	6 (85.7)	143 (78.6)	
Some college	29 (16.6)	1 (14.3)	30 (16.5)	
College	5 (2.8)	0 (0.0)	5 (2.7)	
Post college	4 (2.3)	0 (0.0)	4 (2.2)	
WIC participation^c^				0.53
Yes	163 (92.1)	8 (100.0)	171 (92.4)	
No	14 (7.9)	0 (0.0)	14 (7.6)	
Other children				0.44
Yes	94 (53.1)	5 (62.5)	99 (53.5)	
No	83 (46.9)	3 (37.5)	86 (46.5)	
Previously breastfed^d^				0.05
Yes	89 (93.7)	3 (60.0)	92 (92.0)	
No	6 (6.3)	2 (40.0)	8 (8.0)	
IIFAS total score	67.7±7.2	61.6±8.2	67.4±7.3	0.02
IIFAS score^e^				0.12
Low score	84 (47.5)	6 (75.0)	90 (48.6)	
High score	93 (52.5)	2 (25.0)	95 (51.4)	
Depressive symptoms^f^				0.43
Yes	59 (35.1)	2 (25.0)	61 (34.7)	
No	109 (64.9)	6 (75.0)	115 (65.3)	
Pre-pregnancy BMI^g^	26.0±5.5	27.0±5.4	26.1±5.5	0.61
Pre-pregnancy BMI category^h^				0.27
Normal (< 25)	75 (47.2)	4 (50.0)	79 (47.3)	
Overweight (≥25 or <30)	55 (34.6)	1 (12.5)	56 (33.5)	
Obese (≥30)	29 (18.2)	3 (37.5)	32 (19.2)	

^a^*n* and percentages represent those who answered the question.

^b^ Partner status identified by women and included single but living with a partner.

^c^ Women, Infants and Children.

^d^ Calculated among those who had previous children.

^e^ Low score is less than the median of 68 and high score is equal to or greater than the median.

^f^ Participants scoring positive on at least one of the three depression instruments administered.

^g^ Body Mass Index, calculated in kg/m^2^.

^h^ Centers for Disease Control and Prevention. *Body Mass Index*. Division of Nutrition, Physical Activity and Obesity, Atlanta, GA, 2011. http://www.cdc.gov/healthyweight/assessing/bmi/index.html.

### Any breastfeeding at 6 months

Of 185 women, 135 (73.0%) continued to breastfeed at 6 months postpartum (Table 3). Again, no significant differences were observed in maternal age, country of birth, primary language, years in the U.S., employment status, education level, WIC participation, having other children, presence of depressive symptoms at intake and at 4–6 weeks postpartum, BMI and BMI category between participants who continued to breastfeed at 6 months and participants who did not. Of women who were breastfeeding at 6 months, the majority were partnered (n=116, 86.6%) partnered women were twice as likely to be breastfeeding at 6 months (OR 2.03, p = 0.07) (Table 3). Women who had previously breastfed were much more likely to be breastfeeding at 6 months than women who had not previously breastfed (OR 10.8, p = 0.004). Additionally women who continued to breastfeed at 6 months had significantly higher scores on the IIFAS compared with women who were not (68.2 versus 65.3, p = 0.02) (Table 3).

**Table 3:** Maternal sociodemographic characteristics of participants who were breastfeeding at 6 months postpartum versus those who were not breastfeeding at 6 months postpartum

**Characteristic**	**Breastfeeding at 6 months n (%) or mean** ± **SD (n** = **135)**	**Not breastfeeding at 6 months n (%) or mean** ± **SD (n** = **50)**	**Total n (%)**^**a **^**or mean** ± **SD (n** = **185)**	**P value**
Maternal age in years	26.6±5.4	25.6±4.6	26.3±5.2	0.25
Country of birth				0.33
USA	9 (6.7)	3 (6.0)	12 (6.5)	
Mexico	72 (53.7)	30 (60.0)	102 (55.4)	
Central America	49 (36.6)	13 (26.0)	62 (33.7)	
Other	4 (3.0)	4 (8.0)	8 (4.3)	
Primary language				0.32
English	10 (7.4)	2 (4.0)	12 (6.5)	
Spanish	125 (92.6)	48 (96.0)	173 (93.5)	
Years living in the US	6.4±6.1	6.4±5.4	6.4±5.9	0.97
≤1	22 (17.6)	3 (6.5)	25 (14.6)	
> 1 and ≤ 5	43 (34.4)	23 (50.0)	66 (38.6)	
> 5 and ≤10	38 (30.4)	11 (23.9)	49 (28.7)	
> 10	22 (17.6)	9 (19.6)	31 (18.1)	
Marital status^b^				0.07
Partnered	116 (86.6)	38 (76.0)	154 (83.7)	
Not partnered	18 (13.4)	12 (24.0)	30 (16.3)	
Any employment				0.14
Yes	41 (30.4)	20 (40.0)	61 (33.0)	
No	94 (69.6)	30 (60.0)	124 (67.0)	
Education				0.32
High school or less	104 (77.6)	39 (81.2)	143 (78.6)	
Some college	22 (16.4)	8 (16.7)	30 (16.5)	
College	4 (3.0)	1 (2.1)	5 (2.7)	
Post college	4 (3.0)	0 (0.0)	4 (2.2)	
WIC participation^c^				0.22
Yes	123 (91.1)	48 (96.0)	171 (92.4)	
No	12 (8.9)	2 (4.0)	14 (7.6)	
Other children				0.47
Yes	73 (54.1)	26 (52.0)	99 (53.5)	
No	62 (45.9)	24 (48.0)	86 (46.5)	
Previously breastfed^d^				0.004
Yes	72 (97.3)	20 (76.9)	92 (92.0)	
No	2 (2.7)	6 (23.1)	8 (8.0)	
IIFAS total score	68.2±7.2	65.3±7.3	67.4±7.3	0.02
IIFAS score^e^				0.15
Low score	62 (45.9)	28 (56.0)	90 (48.6)	
High score	73 (54.1)	22 (44.0)	95 (51.4)	
Depressive symptoms at intake^f^	45 (35.7)	16 (32.0)	61 (34.7)	0.39
Yes	81 (64.3)	34 (68.0)	115 (65.3)	
No				
Depressive symptoms 4–6 weeks postpartum				0.37
Yes	23 (17.6)	7 (14.0)	30 (16.6)	
No	108 (82.4)	43 (86.0)	151 (83.4)	
Pre-pregnancy BMI^g^	25.8±5.0	26.7±6.6	26.1±5.5	0.37
Pre-pregnancy BMI category^h^				0.26
Normal (< 25)	56 (46.6)	23 (48.9)	79 (47.3)	
Overweight (≥25 or <30)	44 (36.7)	12 (25.5)	56 (33.5)	
Obese (≥30)	20 (16.7)	12 (25.5)	32 (19.2)	

^a^ n and percentages represent those who answered the question.

^b^ Partner status identified by women and included single but living with a partner.

^c^ Women, Infants and Children.

^d^ Calculated among those who had previous children.

^e^ Low score is less than the median of 68 and high score is equal to or greater than the median.

^f^ Participants scoring positive on at least one of the three depression instruments administered.

^g^ Body Mass Index, calculated in kg/m^2^.

^h^ Centers for Disease Control and Prevention. *Body Mass Index*. Division of Nutrition, Physical Activity and Obesity, Atlanta, GA, 2011. http://www.cdc.gov/healthyweight/assessing/bmi/index.html.

### Exclusive breastfeeding at 6 months

Of the 135 participants continuing to breastfeed at 6 months postpartum (Table 3), 14 (10.4%) were exclusively breastfeeding (Table 4). No significant differences were found in maternal age, marital status at baseline, employment status, WIC participation, having previously breastfed, presence of depressive symptoms at baseline and at 4–6 weeks postpartum, BMI and BMI category at intake between participants who were exclusively breastfeeding at 6 months and those participants who were not exclusively breastfeeding. Of those exclusively breastfeeding at 6 months, the majority were from Mexico and Central America (92.9%) and none were U.S. born; similarly, the majority (78.6%) spoke Spanish as a first language (Table 4). For foreign-born participants, the average number of years in the US was 9.5 for those exclusively breastfeeding and 6.3 for those not exclusively breastfeeding (p = 0.05) (Table 4). The majority of women supplementing their infants at 6 months had no more than a high school education (81.6%, p < 0.001). The majority of those exclusively breastfeeding were first-time mothers (n = 11, 78.6%) (Table 4). Women who were exclusively breastfeeding at 6 months had significantly higher average scores on the IIFAS compared to women who were not exclusively breastfeeding (71.2 versus 67.0, p = 0.04) (Table 4).

**Table 4:** Maternal sociodemographic characteristics of participants who were exclusively breastfeeding at 6months postpartum versus those who were not exclusively breastfeeding

**Characteristic**	**Exclusive breastfeeding at 6 months n (%) or mean** ± **SD (n**=**14)**	**Supplementation at 6 months n (%) or mean** ± **SD (n**=**161)**	**Total n (%)**^**a **^**or mean** ± **SD (n**=**175)**	**P value**
Maternal age in years	27.6±5.9	26.3±5.2	26.4±5.2	0.36
Country of birth				0.03
USA	0 (0.0)	11 (6.9)	11 (6.3)	
Mexico	4 (28.6)	96 (60.0)	100 (57.5)	
Central America	9 (64.3)	46 (28.8)	55 (31.6)	
Other	1 (7.1)	7 (4.4)	8 (4.6)	
Primary language				0.05
English	3 (21.4)	8 (5.0)	11 (6.3)	
Spanish	11 (78.6)	153 (95.0)	164 (93.7)	
Years living in the US	9.5±12.1	6.3±5.0	6.5±6.0	0.05
≤1	4 (28.6)	20 (13.5)	24 (14.8)	
> 1 and ≤ 5	3 (21.4)	58 (39.2)	61 (37.7)	
> 5 and ≤10	3 (21.4)	43 (29.0)	46 (28.4)	
> 10	4 (28.6)	27 (18.2)	31 (19.1)	
Marital status^b^				0.28
Partnered	13 (92.9)	132 (82.5)	145 (83.3)	
Not partnered	1 (7.1)	28 (17.5)	29 (16.7)	
Any employment				0.48
Yes	4 (28.6)	54 (33.5)	58 (33.1)	
No	10 (71.4)	107 (66.5)	117 (66.9)	
Education				<0.001
High school or less	6 (42.9)	129 (81.6)	135 (78.5)	
Some college	2 (14.3)	26 (16.5)	28 (16.3)	
College	4 (28.6)	1 (0.6)	5 (2.9)	
Post college	2 (14.3)	2 (1.2)	4 (2.3)	
WIC participation^c^				0.31
Yes	12 (85.7)	149 (92.5)	161 (92.0)	
No	2 (14.3)	12 (7.5)	14 (8.0)	
Other children				0.02
Yes	3 (21.4)	89 (55.3)	92 (52.6)	
No	11 (78.6)	72 (44.7)	83 (47.4)	
Previously breastfed^d^				0.76
Yes	3 (100.0)	82 (91.1)	85 (91.4)	
No	0 (0.0)	8 (8.9)	8 (8.6)	
IIFAS total score	71.2±8.0	67.0±7.1	67.4±7.3	0.04
IIFAS score^e^				0.22
Low score	5 (35.7)	81 (50.3)	86 (49.1)	
High score	9 (64.3)	80 (49.7)	89 (50.9)	
Depressive symptoms at intake^f^				0.17
Yes	6 (50.0)	49 (31.8)	55 (33.1)	
No	6 (50.0)	105 (68.2)	111 (66.9)	
Depressive symptoms 4–6 weeks postpartum				0.28
Yes	1 (7.1)	28 (17.7)	29 (16.9)	
No	13 (92.9)	130 (82.3)	143 (83.1)	
Pre-pregnancy BMI^g^	24.4±6.5	26.3±5.5	26.1±5.6	0.27
Pre-pregnancy BMI category^h^				0.20
Normal (< 25)	9 (69.2)	65 (44.8)	74 (46.8)	
Overweight (≥25 or <30)	2 (15.4)	51 (35.2)	53 (33.5)	
Obese (≥30)	2 (15.4)	29 (20.0)	31 (19.6)	

^a^ n and percentages represent those who answered the question.

^b^ Partner status identified by women and included single but living with a partner.

^c^ Women, Infants and Children.

^d^ Calculated among those who had previous children.

^e^ Low score is less than the median of 68 and high score is equal to or greater than the median.

^f^ Participants scoring positive on at least one of the three depression instruments administered.

^g^ Body Mass Index, calculated in kg/m^2^.

^h^ Centers for Disease Control and Prevention. *Body Mass Index*. Division of Nutrition, Physical Activity and Obesity, Atlanta, GA, 2011. http://www.cdc.gov/healthyweight/assessing/bmi/index.html.

### Any breastfeeding at 12 months

Of 177 women who initiated breastfeeding, 67 (37.9%) participants continued to breastfeed at 12 months postpartum (Table 5). No significant differences in maternal age, country of birth, primary language, years in the U.S., marital status at intake, employment status, WIC participation, having other children, presence of depressive symptoms at intake and at 4–6 weeks postpartum, IIFAS score, pre-pregnancy BMI and BMI category, and one year postpartum BMI and BMI category between participants who continued to breastfeed at 1 year and those who did not. Of women who were breastfeeding at 12 months, the majority were partnered (n=59, 88.1%) at intake and at 12 months (n=60, 90.1) (Table 5); partnered women were more likely to be breastfeeding at 12 months (OR 2.3, p = 0.07). Of those women breastfeeding at 1 year, 10.4% had a college degree or more while only 2% of women not breastfeeding had a college degree or more (p = 0.06). Of women breastfeeding at 1 year, 36 (100%) had previously breastfed another child.

**Table 5:** Maternal sociodemographic characteristics of participants who were breastfeeding at 1 year postpartum versus those who were not breastfeeding at 1 year postpartum

**Characteristic**	**Breastfeeding at 1 year n (%) or mean**±**SD (n** = **67)**	**No breastfeeding at 1 year n (%) or mean**±**SD (n** = **103)**	**Total n (%)**^**a **^**or mean**±**SD (n** = **170)**	**P Value**
Maternal age in years	27.0±5.2	26.2±5.3	26.5±5.3	0.30
Country of birth				0.19
USA	4 (6.1)	7 (6.8)	11 (6.5)	
Mexico	35 (53.0)	63 (61.2)	98 (58.0)	
Central America	26 (39.4)	27 (26.3)	53 (31.4)	
Other	1 (1.5)	6 (5.8)	7 (4.1)	
Primary language				0.23
English	6 (9.0)	5 (4.9)	11 (6.5)	
Spanish	61 (91.0)	98 (95.1)	159 (93.5)	
Years living in the US	6.4±6.9	6.7±5.4	6.6±6.0	0.76
≤1	11 (17.7)	11 (11.6)	22 (14.0)	
> 1 and ≤ 5	24 (38.7)	36 (37.9)	60 (38.2)	
> 5 and ≤10	17 (27.4)	28 (29.5)	45 (28.7)	
> 10	10 (16.1)	20 (21.0)	30 (19.1)	
Marital status at intake^b^				0.27
Partnered	59 (88.1)	85 (83.3)	144 (85.2)	
Not partnered	8 (11.9)	17 (16.7)	25 (14.8)	
Marital status at 1 year				0.07
Partnered	60 (90.9)	83 (81.4)	143 (85.1)	
Not partnered	6 (9.1)	19 (18.6)	25 (14.9)	
Any employment				0.21
Yes	18 (26.9)	35 (34.0)	53 (31.2)	
No	49 (73.1)	68 (66.0)	117 (68.8)	
Education				0.06
High school or less	52 (77.6)	82 (82.0)	134 (80.2)	
Some college	8 (11.9)	16 (16.0)	24 (14.4)	
College	4 (6.0)	1 (1.0)	5 (3.0)	
Post college	3 (4.5)	1 (1.0)	4 (2.4)	
WIC participation^c^				0.23
Yes	64 (95.5)	94 (91.3)	159 (93.5)	
No	3 (4.5)	9 (8.7)	12 (7.1)	
Other children				0.55
Yes	36 (53.7)	55 (53.4)	91 (53.5)	
No	31 (46.3)	48 (46.6)	79 (46.5)	
Previously breastfed ^d^				0.03
Yes	36 (100.0)	48 (87.3)	84 (92.3)	
No	0 (0.0)	7 (12.7)	7 (7.7)	
IIFAS total score	68.4±7.2	66.6±7.4	67.3±7.4	0.11
IIFAS score^e^				0.05
Low score	28 (41.8)	58 (56.3)	86 (50.6)	
High score	39 (58.2)	45 (43.7)	84 (49.4)	
Depressive symptoms at intake^f^				0.45
Yes	22 (34.9)	32 (32.7)	54 (33.5)	
No	41 (65.1)	66 (67.3)	107 (66.5)	
Depressive symptoms 4–6 weeks postpartum				0.40
Yes	12 (18.5)	16 (15.7)	28 (16.8)	
No	53 (81.5)	86 (84.3)	139 (83.2)	
BMI^g^				0.94
Pre-pregnancy	25.9±5.5	26.0±5.7	26.0±5.6	
1 year	28.6±6.1	29.0±6.5	28.8±6.3	0.73
Pre-pregnancy BMI category^h^				0.89
Normal (< 25)	28 (47.5)	45 (47.9)	73 (47.7)	
Overweight (≥25 or <30)	19 (32.2)	33 (35.1)	52 (34.0)	
Obese (≥30)	12 (20.3)	16 (17.0)	28 (18.3)	
One year BMI category				0.66
Normal (< 25)	18 (28.1)	30 (30.6)	48 (29.6)	
Overweight (≥25 or <30)	24 (37.5)	30 (30.6)	54 (33.3)	
Obese (≥30)	22 (34.4)	38 (38.8)	60 (39.0)	

^a^ n and percentages represent those who answered the question.

^b^ Partner status identified by women and included single but living with a partner.

^c^ Women, Infants and Children.

^d^ Calculated among those who had previous children.

^e^ Low score is less than the median of 68 and high score is equal to or greater than the median.

^f^ Participants scoring positive on at least one of the three depression instruments administered.

^g^ Body Mass Index, calculated in kg/m^2^.

^h^ Centers for Disease Control and Prevention. *Body Mass Index*. Division of Nutrition, Physical Activity and Obesity, Atlanta, GA, 2011. http://www.cdc.gov/healthyweight/assessing/bmi/index.html.

### Multivariate logistic regression models

For breastfeeding initiation, participants who had previously breastfed were 8.3 times as likely to initiate breastfeeding as were participants who had not previously breastfed (OR 8.29, p = 0.05) (Table 6). Having other children was not associated with breastfeeding initiation (OR 0.14, p = 0.072). Additionally, the IIFAS score was not associated with breastfeeding initiation (OR 1.10, p = 0.069). At 6 months, participants who had previously breastfed were much more likely to be breastfeeding compared with participants who had not previously breastfed (OR 18.34, p = 0.01) (Table 6). Participants with other children were less likely to be breastfeeding at 6 months (OR 0.08, p = 0.023). The IIFAS score (OR 1.05, p = 0.065) and marital status (OR 1.69, p = 0.235) were not associated with breastfeeding at 6 months. For women who were *exclusively* breastfeeding at 6 months, women with a college education or more were significantly more likely to be exclusively breastfeeding (college, OR 58.67, p = 0.001; post college, OR 11.35, p = 0.07) than women with less than college education (Table 6). No association was found between the IIFAS score, having other children, marital status and exclusive breastfeeding at 6 months. At 1 year, none of the factors significant in the bivariate analyses were significant in a multivariate model (Table 6).

**Table 6:** Result of multivariate logistic regression analysis of breastfeeding initiation, breastfeeding at 6 months, exclusive breastfeeding at 6 months and breastfeeding at 1 year

**Predictor**^**a**^	**Breastfeeding initiation Adjusted OR (95% CI) (n = 185)**	**Breastfeeding at 6 months Adjusted OR (95% CI) (n = 185)**	**Exclusive breastfeeding at 6 months Adjusted OR (95% CI) (n = 175)**	**Breastfeeding at 1 year Adjusted OR (95% CI) (n = 170)**
Previous breastfeeding				
Yes	8.29 (1.00-68.40)	18.34 (2.01-167.24)^b^		0.00 (0.00-0.00)
No	1.00	1.00		1.00
IIFAS total score	1.1 (0.99-1.22)	1.05 (1.00-1.10)	1.04 (0.94-1.15)	
IIFAS score^c^				
Low score				1.00
High score				1.66 (0.85-3.25)
Other children^d^				
Yes	0.14 (0.02-1.19)	0.08 (0.01-0.70)^e^	0.33 (0.07-1.48)	0.00 (0.00-0.00)
No	1.00	1.00	1.00	1.00
Marital status^f^				
Partnered		1.69 (0.71-4.04)		1.91 (0.70-5.22)
Not partnered		1.00		1.00
Primary language				
Spanish			1.00	
English			0.69 (0.06-8.10)	
Education				
High school or less			1.00	1.00
Some college			1.43 (0.26-7.77)	0.85 (0.33-2.18)
College			58.67 (4.97-692.08)^g^	6.07 (0.65-57.02)
Post college			11.35 (0.84-153.96)	4.78 (0.47-48.93)

^a^ Variables significant at alpha <0.10 were added to the logistic regression analyses.

^b^ P < 0.05.

^c^ Low score is less than the median of 68 and high score is equal to or greater than the median.

^d^ Other children was forced into the regression in order to control for the confounding relationship between past breastfeeding and breastfeeding initiation and duration.

^e^ P < 0.05.

^f^ Marital status at one year analysis was the participant’s marital status at that time and not at intake.

^g^ P < 0.01.

## Discussion

In our group of Latina women, nearly all (177/185, 95.7%) initiated breastfeeding, a rate considerably higher than the national average (80.3%) for Hispanic or Latino mothers [12] and higher than the rate in California for Latinas [14]. At 6 months postpartum, 73.0% (n=135) continued to breastfeed which is more than the national average of 46.0% in Hispanic and Latino mothers [12]. And at 12 months, 37.9% (n = 67) continued to breastfeed, still greater than the national average (24.7%) in Hispanic and Latino mothers [12]. Thus with the exception of exclusive breastfeeding at 6 months, for which no national data are available in this population, our sample had higher rates of breastfeeding at every time interval. However, breastfeeding initiation rates, exclusive breastfeeding rates at 3 months, and breastfeeding rates at 6 months in the state of California are among the highest in the nation and thus may not be specific to this group [40].

Our results reveal that women who had previously breastfed were more likely to initiate breastfeeding, breastfeed at 6 months postpartum and breastfeed at 12 months postpartum. Previous breastfeeding experience has been correlated with the intention to breastfeed [41] and breastfeeding mothers have also been reported to be more likely to have had prior experience breastfeeding [42]. Our data suggest prior breastfeeding experience is also associated with actual breastfeeding initiation and duration. Thus it may be especially important to target first time mothers for breastfeeding promotion as they may be more likely to also breastfeed future children.

In bivariate analyses of exclusive breastfeeding at 6 months postpartum, factors that could be considered a measure of acculturation including country of birth, first language and years in the US were statistically significant. Within foreign-born participants, the majority of those exclusively breastfeeding were from Mexico and Central America (92.9%, n = 13) and spoke Spanish as a first language (78.6%, n = 11) (Table 4). Surprisingly, the mean number of years in the US was *higher* in exclusively breastfeeding group in contrast to prior reports suggesting that the longer Hispanic women live in the US, the shorter the duration of exclusive or any breastfeeding [14,18,19].

A higher score on the Iowa Infant Feeding Attitude Scale is indicative of favorable attitudes toward breastfeeding. Women who initiated breastfeeding, breastfed at 6 months, and exclusively breastfed at 6 months had a higher average score on the IIFAS than their non-breastfeeding counterparts, although the results were not statistically significant, once adjusting for confounders. Previous studies with other population groups have shown that breastfeeding mothers had significantly higher scores, favoring breastfeeding compared with mothers who chose to formula feed, indicating that the IIFAS validly predicts feeding method in some groups [30]. For example, one study initiated in a large urban hospital in Australia found that women with higher IIFAS scores favoring breastfeeding had a longer duration of breastfeeding than women with lower scores favoring formula feeding [44]. Another study with the same population group found that the odds of breastfeeding at hospital discharge increased with increasing total attitude score [23]. In another study of low-income women in Glasgow, comparing women whose attitude scores are 10 units apart, the predicted odds of intended breastfeeding were 3.18 times the odds of intended formula feeding [25]. In addition, maternal infant feeding attitude has previously been shown to be a stronger independent predictor of breastfeeding initiation than sociodemographic factors [22,26].

While we did not validate the IIFAS in the Latino population, this is the first report of the IIFAS used to describe breastfeeding in Latina women. The IIFAS was originally validated in a population of primarily White, middle-class, English-speaking women. Other studies using the IIFAS in Scotland, Australia and Romania have reported adequate predictive validity and internal consistency, and the scale has been previously translated into Romanian and Chinese. To our knowledge, the IIFAS has not been validated in other primarily immigrant populations. In our study, breastfeeding mothers had significantly higher total attitude scores, favoring breastfeeding, compared with mothers not breastfeeding at all points in time except for 1 year postpartum. However, as indicated, these results were not significant in multivariate analysis once potential confounders were taken into consideration.

### Limitations

This study has several limitations inherent in its design. As an observational, prospective cohort study, we can only identify associations, not causal relationships, between maternal sociodemographic factors and attitudes towards breastfeeding and the outcomes of interest. Additionally, the study used a convenience sample recruited in the San Francisco Bay Area and thus the results may not be generalized to all Latino populations in the U.S. and abroad.

Since this was a secondary analysis and the initial study was designed to investigate the association between maternal depression and child weight gain, available data relied upon how questions were asked to participants. More detailed responses would have been preferable for potentially significant factors such as distinguishing high school education from elementary level or no education.

In multivariate analyses, sociodemographic factors were not associated with breastfeeding at one year. Confidence intervals throughout the multivariate models were large, suggesting a low level of precision and the need for a larger sample size. Data were missing at the one year time point that could not be collected through chart review, which may have provided a more robust set of data for analysis. We did not use any instrument to assess acculturation [44] but rather used proxies of acculturation status such as language use and years in the United States; future studies might use these indicators in addition to validated instruments to assess maternal acculturation.

Lastly this was not a validation study of the IIFAS, but only described the association between attitudes surveyed in the IIFAS and actual breastfeeding initiation and duration. Future studies should validate the IIFAS for Latina women.

## Conclusions

Understanding the sociodemographic factors that may influence breastfeeding initiation and duration in Latinas is important as this population continues to grow in the U.S. The ability to appropriately target interventions so that Latina mothers initiate breastfeeding and continue to breastfeed as recommended by the AAP, despite length of time in the U.S., is important in assuring optimal future health of both Latino children and mothers. Specific interventions should target new mothers, as initial feeding decisions will likely be carried over to subsequent children. Additionally, mothers with children at home have specific challenges and need to be targeted for intervention to ensure that breastfeeding continues until 6 and 12 months of age. The IIFAS scale may be a useful tool in predicting breastfeeding outcomes for Latina mothers but further studies are needed to assess the validity of this instrument with this specific population.

## Competing interests

The authors declare that they have no competing interests.

## Authors’ contributions

The study was designed in collaboration with JMW and MBH. KEH and JMW collected all data with assistance from research assistants. KEH shared the main responsibility for statistical analyses with MCW. KEH wrote the manuscript with support from JMW and MBH. All authors read and approved the final manuscript.
